# Validating accelerometry-derived proxies of energy expenditure using the doubly labelled water method in the smallest penguin species

**DOI:** 10.1242/bio.055475

**Published:** 2021-04-01

**Authors:** G. J. Sutton, J. A. Botha, J. R. Speakman, J. P. Y. Arnould

**Affiliations:** 1 School of Life and Environmental Sciences, Faculty of Science & Technology, Deakin University, 221 Burwood Highway, Burwood, VIC 3125, Australia; 2 Marine Apex Predator Research Unit (MAPRU), Institute for Coastal and Marine Research, Nelson Mandela University, Port Elizabeth 6031, South Africa; 3 Institute of Environmental and Biological Sciences, University of Aberdeen, Aberdeen AB24 2TZ, UK; 4 Center for Metabolism, Reproduction and Aging, Shenzhen Institutes of Advance Technology, Chinese Academy of Sciences, Shenzhen, China

**Keywords:** Energy expenditure, Doubly labelled water, Penguin, Accelerometer, Time-energy budget, Bio-logging

## Abstract

Understanding energy use is central to understanding an animal's physiological and behavioural ecology. However, directly measuring energy expenditure in free-ranging animals is inherently difficult. The doubly labelled water (DLW) method is widely used to investigate energy expenditure in a range of taxa. Although reliable, DLW data collection and analysis is both financially costly and time consuming. Dynamic body acceleration (e.g. VeDBA) calculated from animal-borne accelerometers has been used to determine behavioural patterns, and is increasingly being used as a proxy for energy expenditure. Still its performance as a proxy for energy expenditure in free-ranging animals is not well established and requires validation against established methods. In the present study, the relationship between VeDBA and the at-sea metabolic rate calculated from DLW was investigated in little penguins (*Eudyptula minor*) using three approaches. Both in a simple correlation and activity-specific approaches were shown to be good predictors of at-sea metabolic rate. The third approach using activity-specific energy expenditure values obtained from literature did not accurately calculate the energy expended by individuals. However, all three approaches were significantly strengthened by the addition of mean horizontal travel speed. These results provide validation for the use of accelerometry as a proxy for energy expenditure and show how energy expenditure may be influenced by both individual behaviour and environmental conditions.

## Abstract (precis)

**Summary:** This paper investigates and validates the use of animal-borne accelerometers to measure energy expenditure in the smallest penguin species.

## INTRODUCTION

Energy is a finite resource and a central currency in determining the behaviour and physiology of animals (Butler et al., 2004; Speakman and Król, 2010). How animals allocate their time and energy critically influences important aspects of their life history, including food acquisition, growth and reproduction (McNamara and Houston, 1996). Accurately estimating the energetic costs of these behaviours has long been a central theme in behavioural ecology and is crucial to understanding how animals adapt to environmental variability. However, directly measuring the energy expenditure (DEE) of free-ranging animals is inherently difficult due to various logistical constraints (Speakman and Racey, 1988).

Techniques that measure the energy expended by free-ranging animals have centred around three methods. (1) Determining time-activity budgets and assigning energy values to observed activities (Utter and LeFebvre, 1973; Weathers and Nagy, 1980); (2) estimating energy expenditure from the relationship between heart rate and CO_2_ production through implanted heart rate loggers (Arnold et al., 2006; Butler et al., 2004; Green et al., 2001; Halsey et al., 2008) and; (3) the measuring washout rates of injected stable isotopes through the doubly labelled water method (DLW) (Speakman, 1993). Each technique is associated with a suite of drawbacks, namely accuracy (Goldstein, 1988), ability to calibrate measurements on captive populations (Goldstein, 1988; Morrier and McNeil, 1991), invasiveness (Green, 2011) and cost of analyses (Butler et al., 2004; Speakman, 1997).

Of the aforementioned techniques, the DLW method requires only blood samples at the beginning and end of the measurement period (Speakman and Racey, 1988). It can thus be more easily applied to free-ranging animals. However, the DLW method only provides a single energy expenditure value over the measurement period and the financial cost of isotopes and their analyses may limit the size and number of animals that can be sampled (Butler et al., 2004; Shaffer, 2011). Therefore, it is important to develop and validate techniques to measure energy expenditure over greater temporal periods.

Over the past two decades, there has been widespread deployment of animal-borne accelerometer data loggers. (Brown et al., 2013; Vacquié-Garcia et al., 2015; Yoda et al., 2001). This high resolution data can be used to infer the behavioural states and fine-scale activity budgets of free-ranging individuals (Battaile et al., 2015; Collins et al., 2016). These devices provide whole body acceleration and, with increasing battery life, can provide information over various spatial and temporal scales (Brown et al., 2013; Vacquié-Garcia et al., 2015; Yoda et al., 2001). With increasing miniaturisation of accelerometer data loggers, it is now possible to obtain this information for relatively small animals (i.e. <100 g) over extended periods (Brown et al., 2013; Hammond et al., 2016).

In addition to providing information on behavioural activity, accelerometry can used to quantify energy expenditure (Green et al., 2009; Wilson et al., 2006). By correlating accelerometry-derived estimates of energy expenditure with traditional techniques, the need for such highly-invasive, costly and/or labour-intensive methods of estimating energy expenditure may be by-passed in the future (Halsey et al., 2011). Simple predictive correlations between Overall and Vectorial dynamic body acceleration (e.g. ODBA and VeDBA, respectively) and energy expenditure concurrently measured using the DLW method have shown varying degrees of success (Elliott et al., 2013; Hicks et al., 2020; Pagano and Williams, 2019). Such relationships have been improved somewhat by separating acceleration into behavioural components (Elliott et al., 2013; Jeanniard-du-Dot et al., 2017a). However, studies addressing the relationship between energy expenditure derived from DLW methods and accelerometers have been largely limited to captive or pseudo-captive animals (but see: Elliott et al., 2013; Hicks et al., 2020; Jeanniard-du-Dot et al., 2017a).

The little penguin (*Eudyptula minor*), the smallest penguin species, is distributed in colonies around the southern coast of Australia and New Zealand (Chiaradia et al., 2007). The majority of the population is concentrated in south-eastern Australia, a region of rapid oceanic warming (Crossin et al., 2014; Lough and Hobday, 2011; Mickelson et al., 1991). The anticipated changes in the marine ecosystem are likely to impact the distribution and abundance of prey for the little penguin (Berlincourt and Arnould, 2015; Poloczanska et al., 2007), potentially causing them to work harder (i.e. expend more energy) during foraging. Therefore, an ability to efficiently quantify energy expenditure in free-ranging little penguins is crucial to understanding how an individual's effort may change in response in prey availability (Barbraud et al., 2012; Crossin et al., 2014). While accelerometry is increasingly being used to investigate the foraging behaviour of penguins (Carroll et al., 2016; Kokubun et al., 2011; Van Dam et al., 2002), few have addressed the predictive ability of accelerometers for estimating energy expenditure in free-ranging individuals (Hicks et al., 2020).

Little penguins are diurnal foragers, leaving and returning to the colony at sunrise and sunset, respectively (Klomp and Wooller, 1991). Due to their relatively short foraging trip durations throughout the breeding season (Chiaradia and Kerry, 1999) and their small body size (purporting low dosage requirements of DLW), little penguins make an ideal model species for investigating accelerometry-derived estimates of at-sea energy expenditure in aquatic endotherms. The aims of the present study, therefore, were to determine what methods are useful in determining energy expenditure of free-ranging little penguins. The accuracy of three accelerometry-derived estimates of energy expenditure: (1) vectorial dynamic body acceleration (VeDBA); (2) activity-specific body acceleration; and (3) time-activity budgets; were compared to those measured by the DLW method.

## RESULTS

A total of 36 individuals were dosed and instrumented for the study (Gabo Island; GI: 15, London Bridge; LB: 21). However, device malfunction resulted in 11 individuals without accelerometer/dive data. In addition, four individuals from GI returned from their foraging trip with blood isotopic levels too close to background levels for accurate measures of at-sea energy expenditure to be determined. 12 individuals remained on land following injection of DLW and these individuals were used to determine daily energy expenditure on land (DEE_DLW-L_, Table 1).

**Table 1.**
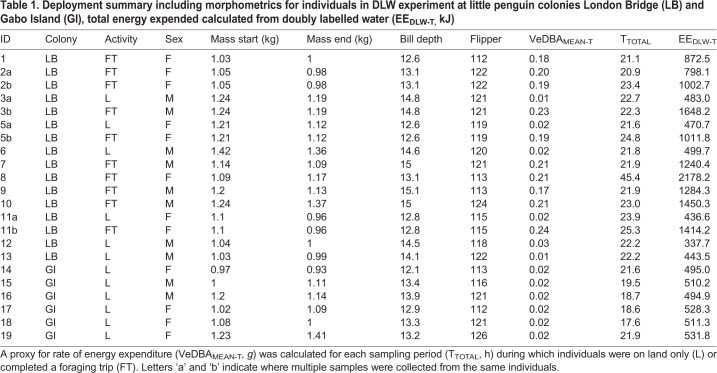
**Deployment summary including morphometrics for individuals in DLW experiment at little penguin colonies London Bridge (LB) and Gabo Island (GI), total energy expended calculated from doubly labelled water (EE_DLW-T,_ kJ)**

Mass specific at-sea metabolic rate (DEE_DLW-S_ kJ kg^−1^ d^−1^) was obtained over a single foraging trip for eight individuals and over two foraging trips for one individual with blood samples collected between foraging trips (*N*=10; Table 2). Individual eight completed two 1-day foraging trips, returning in the early hours of the morning before sunrise and leaving again without a blood sample being collected (Table 2). As there was only one repeated sampling period at sea, standard linear models were used to determine relationships.

**Table 2.**
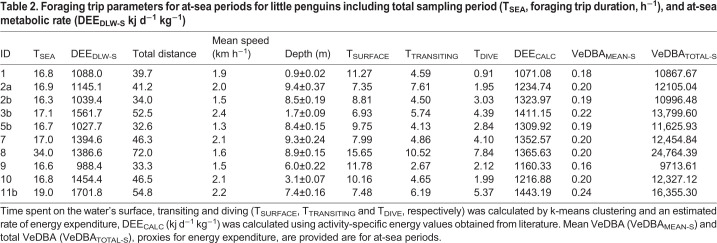
**Foraging trip parameters for at-sea periods for little penguins including total sampling period (T_SEA_, foraging trip duration, h^−1^), and at-sea metabolic rate (DEE_DLW-S_ kj d^−1^ kg^−1^)**

The body mass of individuals at sampling prior to departure on a foraging trip and after returning was 1.14±0.03 kg and 1.10±0.04 kg, respectively. Foraging trips lasted on average 19.3±1.5 h during which individuals covered total horizontal distances of 47±4.2 km. Individuals performed 369±25 dives and covered total vertical distances of 6.3±1.7 km, during 18±2 dives h^−1^ to an average depth of 7.5±1.0 m. Body mass differed significantly between the sexes (t_8_=3.2, *P*<0.01) but there were no sex differences apparent for flipper length, dive depth, foraging range, foraging trip duration and mean or total VeDBA (*P*>0.05 in all cases).

The DEE_DLW-L_ values ranged 350.3–580.5 kJ kg^−1^ d^−1^ and there were no statistical differences between the sexes (t_6_=0.5, *P*>0.05). The calculated rate of daily energy expenditure at sea (DEE_DLW-S_) was significantly greater (1392.4±119.6 kJ kg^−1^ d^−1^) than on land (429.1±16.1 kJ kg^−1^ d^−1^; t_8_=10.4, *P*<0.001), and did not differ between the sexes (*P*>0.05 in both cases). These values then provided the relationships between DEE_DLW-S_ and the three accelerometry derived indices of energy expenditure.

### Approach 1

**Fig. 1.**
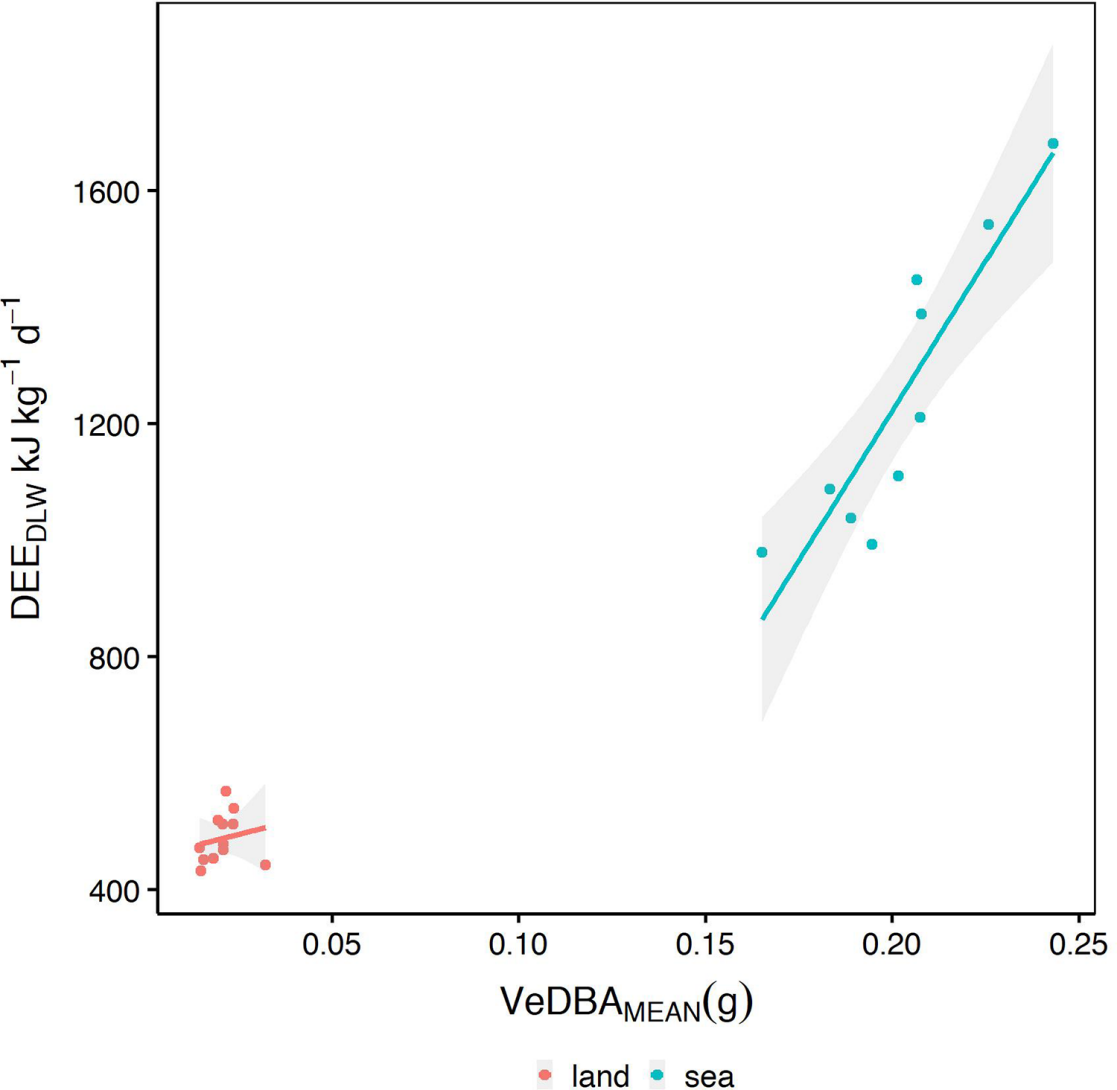
**Relationships between rate of energy expenditure derived from doubly labelled water DEE_DLW_ (kJ kg^−1^ d^−1^) and mean VeDBA (VeDBA_MEAN_ g), a proxy for energy expenditure, calculated for periods on land and at sea.**

Significant differences were evident in the mean VeDBA obtained for when the animal was on land (VeDBA_MEAN-L;_ 0.02±0.01 g) and mean VeDBA for when the animal was at sea (VeDBA_MEAN-S_; 0.2±0.01 g, t_8_=29.8, *P*<0.001). While there was a weak relationship (r^2^<0.5) between VeDBA_MEAN-L_ and DEE_DLW-L_ (*r*^2^=0.13; Fig. 1), there was a positive significant relationship between DEE_DLW-S_ and VeDBA_MEAN-S_ (*r*^2^=0.82, *F*_1,8_=32.12, *P*<0.001; Fig. 4a) giving the relationship:(1)
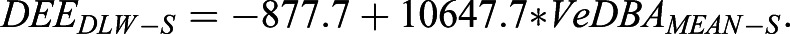

Model selection after additional predictor variables were added resulted in the most parsimonious model for predicting DEE_DLW-S_ (kJ kg^−1^ d^−1^) including the predictor variables VeDBA_MEAN-S_ (*g*) and mean speed (MS, km h^−1^; Table S2). The addition of MS to the equation provided an improved predictive relationship (*r*^2^=0.84; Table 4, Fig. 4b):(2)
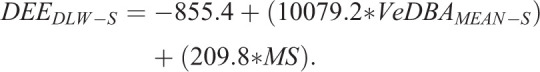


### Approach 2

**Fig. 2.**
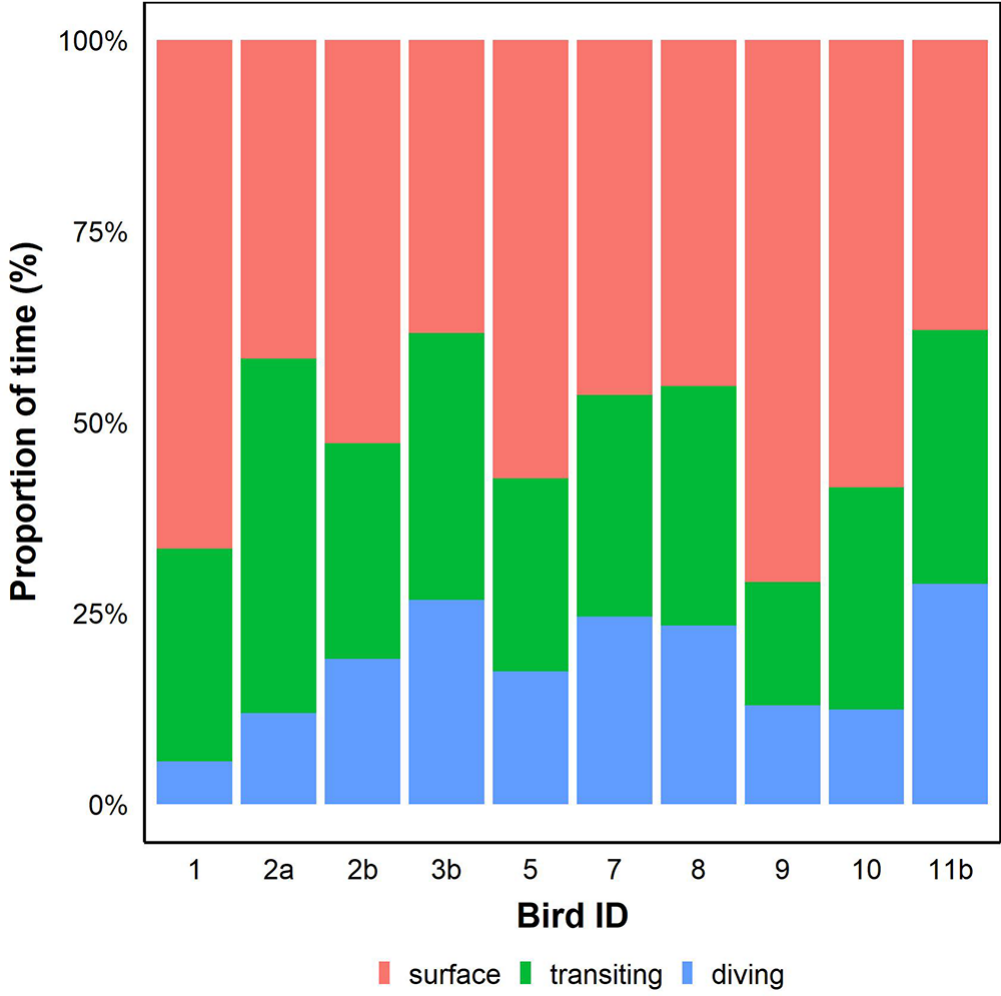
**Proportion of time spent diving, transiting and resting on the sea surface for little penguins.**

**Fig. 3.**
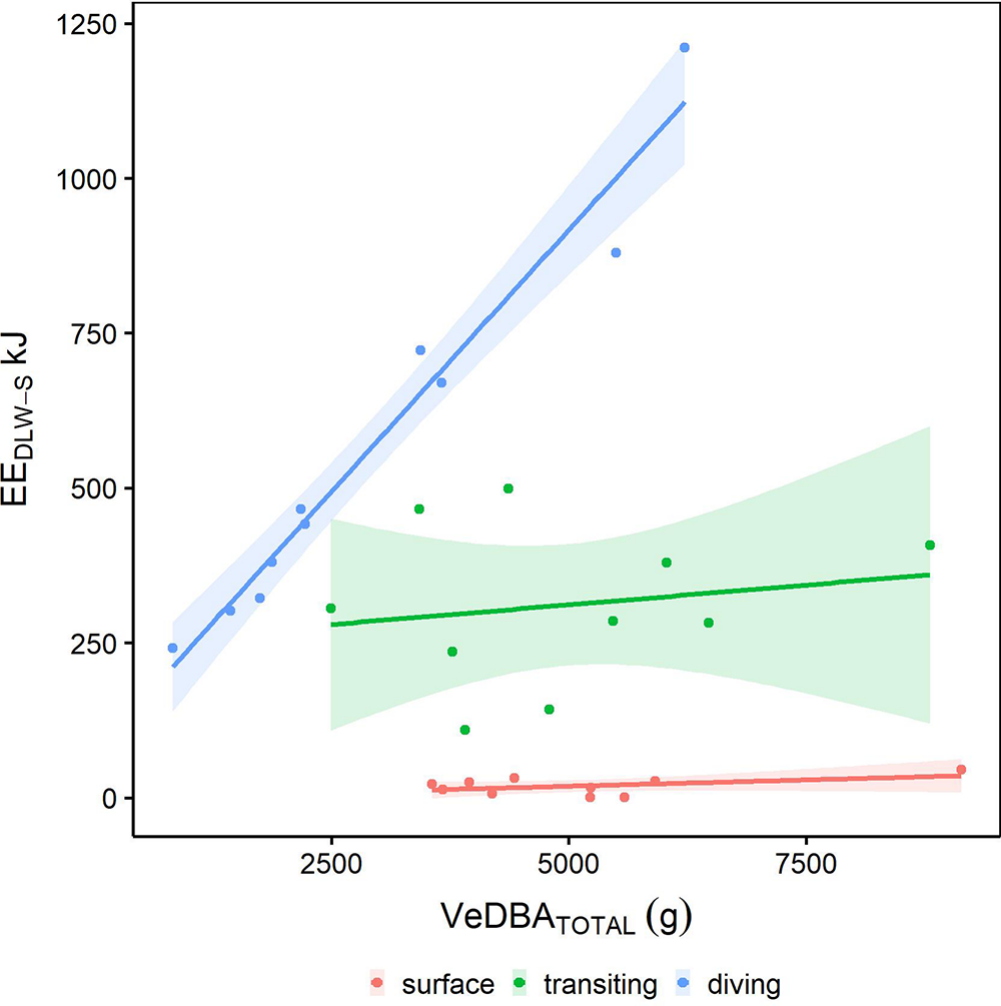
**Estimates of activity-specific energy expenditure for diving, transiting and sea-surface resting.** Plot shows the predicted model linear regression (solid line) and 95% confidence intervals for the relationship between at-sea energy expenditure determined from doubly labelled water (EE_DLW-S_) and VeDBA, a proxy for energy expenditure, for each activity. Regression equations and r^2^ statistics for sea-surface resting, transiting and diving are EE_DLW-S(SURFACE)_=0.005 * VeDBA_SURFACE_+5.7, *r*^2^=0.91; EE_DLW-S(TRANSIT)_=0.01*VeDBA_TRANSIT_ +248.2, *r*^2^=0.03; and EE_DLW-S(DIVE)_=0.17 * VeDBA_DIVE_ +72.6, *r*^2^=0.93, respectively.

Activity budget analysis revealed the highest proportion of time at-sea was spent on performing surface activities (50.5±3.6%), followed by transiting (29.4±2.3%; Fig. 2) and diving behaviour (17.8±2.3%). Mean VeDBA for sea-surface resting was the lowest (0.15±0.01 g) while transiting and diving were similar (0.24±0.01 g and 0.23±0.01 g, respectively). Activity-specific VeDBA values were compared with the activity estimates determined from Eq. 8. Correlations between total VeDBA and total predicted energy expended were found for sea-surface resting (*r*^2^=0.91, *F*_1,8_=89.3, *P*<0.001) and diving (*r*^2^=0.96, *F*_1,8_=207.4, *P*<0.001; Fig. 3). There was no significant relationship found for transiting which was of low predictive accuracy (*r*^2^=0.03, *F*_1,8_=0.3, *P*>0.05).

**Fig. 4.**
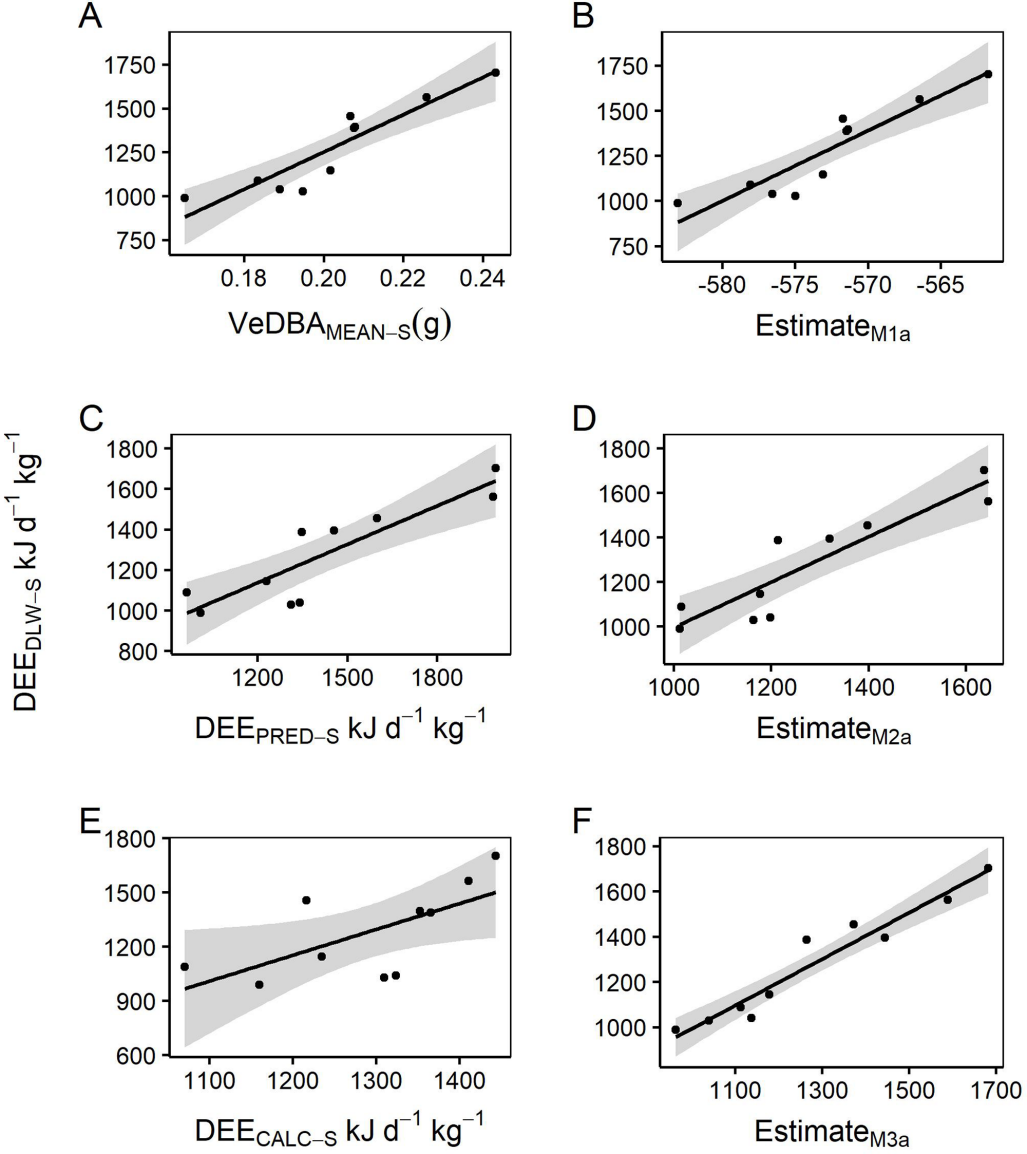
**Correlations between mass-specific at-sea daily energy expenditure (DEE_DLW_) derived from doubly labelled water method and three approaches of estimating energy expenditure.** Approach 1 mean VeDBA [VeDBA_MEAN-S_; A; DEE_DLW-S_=−877.7+(10647.7 * VeDBA_MEAN-S_); *r*^2^=0.82]; Approach 2 activity specific VeDBA [DEE_PRED-S_; C; DEE_DLW-S_=377.1+(0.6 * DEE_PRED-S_); *r*^2^=0.78] and; Approach 3 application of energy estimates derived from previous studies [DEE_CALC-S_; E; DEE_DLW-S_=−566.4+(1.4 * DEE_CALC-S_); *r*^2^=0.44] and the estimates (M1a, M2a and M3a) derived from the most parsimonious models identified through model selection included the additional model parameters mean speed (MS) and mean dive depth (MDD). Equations for these relationships are: B; DEE_DLW-S_=−855.4+(10079.2 * VeDBA_MEAN-S_)+(209.8 * MS); *r*^2^=0.84; D; DEE_DLW-S_=300.8+(0.6 * DEE_PRED-S_)+(259.3 * MS), *r*^2^=0.82 and F; DEE_DLW-S_=−1472.49+(1.7 * DEE_CALC-S_)+(422.7 * MS) +(−42.8 * MDD), *r*^2^=0.93).

Linear modelling of DEE_DLW-S_ and the derived mass-specific predicted rate of energy expenditure (DEE_PRED-S,_ kJ·kg^−1^·d^−1^) revealed a strong positive relationship (*r*^2^=0.78, Fig. 4c):(3)
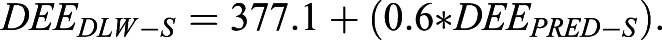


Model selection to determine the most parsimonious model resulted in the addition of Mean Speed (MS, km·h^−1^; Table S3). The inclusion of MS further improved the predictive relationship (*r*^2^=0.82, Fig. 4d; Table 4):(4)
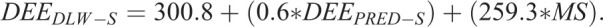

### Approach 3

The calculated average at-sea energy expenditure rate (DEE_CALC-S_) determined from activity specific energy values obtained from literature and applied to time-activity budgets was 1270.2±42.2 kJ d^−1^ kg^−1^. Linear modelling of DEE_DLW-S_ and DEE_CALC-S_ revealed a weak positive relationship (*P*=0.06, *r*^2^=0.44) (Fig. 4E) with the confidence intervals of DEE_CALC-S_ crossing zero (Table 4), indicating this parameter is not a good explanatory variable. Model selection resulted in the most parsimonious model (*r*^2^=0.93, Table 4) for predicting DEE_DLW-S_ including MS (km·h^−1^) and Mean dive depth (MDD, m; Table S4; Fig. 4f):(5)
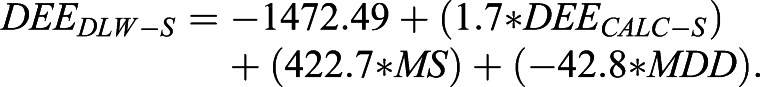


## DISCUSSION

Developing and validating techniques for measuring the metabolic rate of free-ranging animals is central to understanding an animal’s physiological, behavioural and evolutionary ecology (Butler et al., 2004; McNamara and Houston, 1996). Accelerometry and dynamic body acceleration has been used to determine behavioural patterns in a range of taxa, and is increasingly used as a proxy for energy expenditure (Barwick et al., 2018; Hinchcliff et al., 1997; Noda et al., 2014). In the present study, indices of movement (i.e. VeDBA), both in the simple correlation and activity-specific approaches, was shown to be a good predictor of the mass-specific at-sea metabolic rate derived from DLW. The approach using activity-specific energy expenditure values obtained from literature did not accurately reflect the energy expended by individuals in the present study. However, all three approaches were significantly strengthened by the addition of mean horizontal travel speed. These results suggest that proxies of energy expenditure may be influenced by both individual behaviour and environmental conditions.

### Energy expenditure and VeDBA

The estimates of on-land metabolic rate observed in the present study (mean: 429.1 kJ kg^−1^ d^−1^) are within range of the standard metabolic rate (SMR) for captive little penguins and the fasting metabolic rate of free-ranging individuals (426.0 kJ kg^−^1 d^−1^ and 560 kJ kg^−1^ d^−1^, respectively) (Costa et al., 1986; Stahel and Nicol, 1982). On-land periods for little penguins may include energetically expensive activities such as walking, preening, territorial defence and feeding chicks, all of which predominantly occur at night. However, the on-land sampling periods in the present study were comprised mainly of daylight hours, where individuals remain in their nest burrows. Hence, the on-land energy expenditure recorded in the present study is likely to be representative of the physiological processes associated with fasting.

Little movement activity was recorded in the accelerometry values for individuals who remained in their burrows over the sampling period. As accelerometry measures body acceleration and movement (Wilson et al., 2006), it is not surprising that there was a weak relationship between VeDBA_MEAN-L_ and DEE_DLW_-_L_. The range of on-land energy expenditure values derived from DLW was narrow in comparison to the at-sea values as little penguins who stayed ashore during the day remained in their nest burrows to avoid predators (Colombelli-Négrel and Tomo, 2017). This suggests that the variation observed in the on-land metabolic rate of individuals may be attributed to variation in physiological processing such as digestion, thermoregulation and cellular processes not measured by accelerometers.

The average at-sea metabolic rate observed in the present study (1278.8 kJ kg^−1^ d^−1^) was within the range of that reported in previous metabolic studies of free-ranging little penguins (1124–1500 kJ kg^−1^ d^−1^) (Bethge et al., 1997; Costa et al., 1986). The variation observed in the range of daily energy expenditure in the present study may be attributed to a combination of at-sea activity budgets and offspring provisioning. Indeed, little penguins attending to late-stage chicks had maximum daily energy expenditure rates of 2532 kJ kg^−1^ d^−1^ (Gales and Green, 1990), as measured by DLW method, indicating that energetic requirements may increase with chick age. In the present study, it was not possible to sample individuals based on chick age and breeding adults were provisioning chicks at various stages of chick rearing. Therefore, a proportion of the variability in at-sea metabolic rate may be influenced by differences in the energetic demands of resource provisioning.

At-sea variation in daily metabolic rates could also be associated with physiological processes such as food digestion and thermoregulation in water. Energy utilised for the digestion of prey is estimated to be equivalent to 13–15% of the available energy content of the prey in little penguins (Green et al., 2006). Therefore, the at-sea energy expenditure of an individual may be influenced by the amount of prey consumed. While thermoregulation in water is thought to influence the energy expended by little penguins (Stahel and Nicol, 1982), individuals in the present study were sampled over the same periods. As such, the water temperatures experienced by all individuals was assumed to be similar and, therefore, would have had a negligible effect on the individual variations in measured energy expenditure. While it is possible that these factors may influence at-sea energy expenditure, VeDBA_MEAN-S_ was strongly correlated to DEE_DLW-S_, accounting for more than 80% of the variation observed. This suggests that individuals have high locomotive costs, with the costs associated with physiological processes not measured by accelerometry being comparatively small.

In the present study, the relationship between DEE_DLW-S_ and VeDBA_MEAN-S_ was substantially improved by the addition of mean speed as a predictor. Mean speed varied substantially between individuals, with those that travelled at a faster speed having higher rates of energy expenditure. In addition to active movement through the physical medium of water, measurement of mean speed also encompasses passive transport that may be influenced by currents, sea-state and wind conditions that could account for variation in energy expenditure not be adequately captured by accelerometry. Indeed, mean speed and mean dive depth was significantly correlated with DEE_DLW-S_ (*r*^2^=0.64; Table S1) and, as a predictor variable, mean speed improved the models in every investigated approach.

### Activity-specific metabolic rates

Previous studies have attributed weak correlations between body acceleration and energy expenditure measured by DLW to variability in activity levels (Jeanniard-du-Dot et al., 2017a). To overcome this, time-activity budgets can be modelled to obtain activity-specific energetic values which seem to improve these relationships. Over short sampling durations, strong relationships between the rate of energy expenditure and body acceleration in free-ranging marine predators has been reported for individuals performing high-energy activities (Elliott et al., 2013; Stothart et al., 2016). Similarly, in the present study, most individuals undertook foraging trips <24 h and spent more considerable proportions of that time undertaking high-energy activities (i.e. diving and transiting). Hence, the simple correlative and activity-specific approaches were of similar predictive capacity.

Surface activities were less energetically costly than transiting and diving activities, but overall more expensive than the on-land energy expenditure. This could be because surface activities encompass post-dive resting as well as other behaviours such as preening which could be associated with higher costs (Goldstein, 1988; Wooley and Owen, 1978). Diving behaviour was the most expensive at-sea activity for individuals in the present study, at 7.6 times the SMR calculated by Bethge et al. (1997). This is within the range of diving metabolic rates observed in other penguin species (Chappell et al., 1993; Nagy et al., 2001).

Transiting at the surface was less expensive than diving and was equivalent to 1.6 times the SMR (Bethge et al., 1997). Transiting marine vertebrates usually swim at depths three times their body widths, which is thought to reduce drag forces and the cost of transportation (Boyd and Hoelzel, 2002; Hindle et al., 2010). Transiting can vary in speed, and may be attributed various behaviours such as prey capture behaviour, commuting to and from the colony or between foraging patches (Sutton et al., 2020). The fine-scale sea state variation may also influence the energy expended during transiting. Thus, the low correlation between VeDBA and the estimated energy expended during transiting could indicate a combination of energetic variation in this behavioural mode and external factors influencing the energy expended during this activity.

The summation of activity-specific acceleration should be a better predictor of energy expenditure when there is a large difference in energetic costs between different behaviours (Elliott et al., 2013; Laich et al., 2011). The observed at-sea behaviour categories were found to be associated with different VeDBA values resulting in different activity-specific estimates. Consequently, VeDBA was considered a good predictor of energy expenditure using Approach 2. While the mean travelling speed improved the predictive capacity of Approach 2, comparisons of modelling approaches 1 and 2 indicate that they are of similar predictive capacity. However, Approach 2, is more labour intensive with regard to data processing and determining activity budgets than the simple correlative approach (i.e. Approach 1).

In the present study, Approach 3 was unsurprisingly found to be the least effective method for predicting at-sea metabolic rate. This is likely due to the accuracy of activity-specific energy expenditure estimate for this species. Using previously determined estimates of activity-specific energy expenditure may be problematic as individuals in captive environments may be less motivated to perform behaviours similar to those of their free-ranging counterparts. For example, the energetic values for transiting in water recorded for little penguins in laboratory conditions was found to be considerably slower than what was recorded in free-ranging individuals (Bethge et al., 1997). This raises questions with regards to the validity of applying such values to activity budgets of free-ranging individuals.

Numerous studies using activity-budgets (derived from accelerometry or other methods) to estimate energy expenditure in free-ranging animals have been performed using estimates obtained from controlled conditions or from phylogenetically distant species moving in similar locomotory modes and in similar environments (Collins et al., 2016; Goldstein, 1988; Ladds et al., 2018; Shaffer et al., 2004). The poor predictive capacity of Approach 3 in the present study highlights the potential inaccuracy of such studies and the need for accurate species-specific and activity-specific energy expenditure values. Ultimately, without validation, the accuracy and applicability of these methods for use on free-ranging animals remains unknown.

In summary, accelerometry-derived proxies provided an accurate estimation of at-sea energy expenditure measured by the DLW method in little penguins. Activity-specific energy expenditure predicted from a modelling approach was slightly more accurate than a simple correlation approach. However, both relationships were improved with the addition of mean speed as a predictor, indicating that the transport medium may impact both DLW and acceleration measurements. The results of the present study further support the use of accelerometry as a means to estimate energy expenditure in free-ranging animals but emphasises the need for more validation studies. Confirming the strong predictive relationship between energy expenditure and accelerometry may provide greater understanding of how animals respond to shifts in their environment such as the predicted changes habitat and prey availability resultant from warming ocean temperatures in population hot spots.

## MATERIALS AND METHODS

### Study sites and animal handling

The study was conducted at two little penguin colonies in Bass Strait, south-eastern Victoria, Australia: Gabo Island (37.56° S, 149.91° E, GI); and London Bridge (38.62° S, 142.93° E, LB). Gabo Island, in eastern Bass Strait, which has previously been estimated to host approximately 30–40,000 little penguins (Fullagar et al., 1995), while London Bridge is a small mainland colony in western Bass Strait, which hosted ∼100 individuals during the study period. Data collection occurred during November to December 2018, coinciding with post-guard phase of the breeding season where both adults normally forage at sea during the day and return most nights to feed their offspring.

Measurements of daily energy expenditure for adult breeding little penguins were obtained using the DLW method. Individuals were captured at their nest burrow, placed in a cloth bag and weighed using a spring balance (±10 g, Super Samson, Salter Brecknall, UK). An initial blood sample (<1 ml) was collected by venipuncture of a tarsus vein into a heparinised syringe to establish background levels of ^2^H and ^18^O (method D; Speakman and Racey, 1988). Individuals were then administered an intramuscular injection [1.01±0.03 g of DLW (64.3% ^18^O and 34.1% ^2^H)]. Syringes were weighed before and after injection to calculate the mass of DLW injected into each bird (±0.001 g, FX300i milligram balance, A&D Company Ltd, Japan). Following injections, penguins were returned to their nest burrow for a mean of 3.36±0.09 h, during which time the isotopes equilibrated with the body water pool (Gales and Green 1990).

After the equilibrium period, individuals were removed from the nest and instrumented with two devices: a GPS (Mobile Action Technology, I-gotU, GT-120, 44.5×28.5×13 mm, 20 g) which sampled location at 1 min intervals; and a combined accelerometer/depth recorder (Gulf Coast Data Concepts 76×46×16 mm, 45 g) which sampled depth and acceleration at 1 and 25 Hz, respectively. The devices were securely attached to the feathers along the lower dorsal midline using waterproof tape (Tesa 4651, Beiersdorf, AG, GmbH, Hamburg). A second blood sample was then collected to establish the isotope equilibrium levels and individuals were returned to their nests.

All nests were monitored in the late afternoon of the next day to determine whether individuals had departed to sea on a foraging trip. If an individual was present, it was weighed and a blood sample was collected to obtain a measure of energy expenditure on land. If the individual was absent, the burrow was monitored during the subsequent night and, when the individual returned, it was recaptured after feeding its chicks, weighed and a blood sample was collected before being released. This process continued for the next 2 days and nights, enabling multiple energy expenditure periods to be sampled in some individuals, before a final blood sample was collected and the data loggers were removed. After the devices were removed, the morphometrics of bill depth, bill length and head length were measured using a Vernier Caliper (±0.1 mm) and flipper length was measured using a ruler (1 mm). Sex was determined from bill depth following the methods of Arnould et al. (2004).

### Data processing and statistical analyses

All blood samples were centrifuged to isolate the plasma from red blood cells within 4 h of collection. Aliquots of plasma were then transferred into flame-sealed capillary tubes (100 µL) until analyses were performed. Isotope enrichment of blood samples was determined by off-axis integrated cavity output spectroscopy (Berman et al., 2012; Melanson et al., 2018). Total body water was estimated from the ^18^O dilution space using the plateau method (Speakman, 1997). Isotope enrichments were converted into estimates of total energy expenditure (*EE_DLW_* kJ) during measurement periods using the two-pool method (Eqn 7.17) (Speakman, 1997).

The GPS location data were filtered to remove erroneous fixes that exceeded the maximum average horizontal travel speed of 7.2 m·s^−1^ (Hoskins et al., 2008), and dive behaviour data obtained from the depth sensor were corrected for depth drift, using the *diveMove* package (Luque, 2007) in the R statistical environment (version 1.1.463) (R Core Team 2018). The filtered GPS track and the dive data were linearly interpolated and merged to the accelerometer data. For each DLW sampling period, the time spent on land and time at sea were calculated from the GPS locations and accelerometry. The foraging trip metrics: range (km), total duration (h), mean speed (km h^−1^) and the dive parameters of mean dive depth (m), total vertical distance travelled (km) were then calculated for each trip using the *trip* and *diveMove* packages, respectively.

At-sea energy expenditure (*EE_DLW-S_* kJ) was calculated by subtracting on land energy expenditure (*EE_DLW-L_* kJ) from the total energy expenditure over the sampling period (*EE_DLW-T_* kJ) using the following equation:(6)
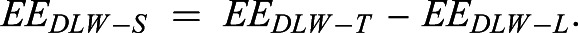


Individual estimates of EEDLW-L were determined from the average rate of energy expenditure obtained from individuals sampled while only on land, and adjusted for the proportion of the foraging trip sample duration on land. The EEDLW-S values were then converted to estimates of mass-specific rate of at-sea energy expenditure (DEEDLW-S kJ·kg−1·d−1) and compared to proxies obtained from the accelerometry data using three methodological approaches.

### Approach 1

Accelerometer data for each sampling period were filtered to separate dynamic acceleration (attributed to animal movement) from static acceleration (reflecting animal position with respect to gravity) using a 1 s running mean and, as accelerometers were not attached to the centre of gravity of the animal, Vectorial Dynamic Body Acceleration (VeDBA) was calculated using the following equation:(7)
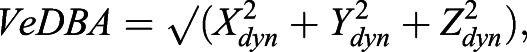
where X, Y and Z are the dynamic acceleration (*dyn*) of horizontal (surge), vertical (heave) and lateral (sway) movements, respectively.

The sum of VEDBA [area under the curve (Ladds et al., 2017), VEDBA_SUM_] and mean VeDBA (VeDBA_MEAN_), proxies for animal movement, over the study period were calculated for each on-land (VeDBA_SUM-L_, VeDBA_MEAN-L_, respectively) and at-sea sampling period (VeDBA_SUM-S_, VeDBA_MEAN-S_, respectively), as determined from GPS locations at the colony and confirmed by accelerometry (i.e. angle of device indicating individual out of the water).

### Approach 2

To determine if activity-specific estimates of energy expenditure provided a better relationship with DEE_DLW-S_ than Approach 1, behavioural categories were identified from the accelerometry and dive data in at-sea periods using *k*-means clustering analysis in the Ethographer package in IgorPro (Wavemetrics Inc., Portland OR, USA, version 6.3.7.2) (Sakamoto et al., 2009). Three behaviour categories were identified: sea-surface resting (e.g. above surface behaviours/grooming on the sea-surface); transiting (horizontal sub-surface movement <2 m); and diving (sub-surface movement >2.5 m). The duration of each of the behaviour categories and the mean and total VeDBA was determined for each individual.

Activity-specific energy expenditure was calculated following the methods of Jeanniard-du-Dot et al. 2017a, 2017b. Parameter estimates for each individual were calculated for the behaviour categories based on time spent in each of the behaviour categories. For each individual, the parameter estimates were added to the following equation:(8)
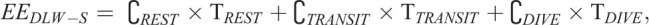
where EE_DLW–S_ is the total at-sea energy expenditure derived from Eqn. 6, C*_i_* is the parameter estimate for the rate of energy expenditure for each activity and T*_i_* is the time spent (h) in each at-sea behaviour category. The resulting linear equations were used to predict total energy expenditure at-sea, which was converted to an estimate of predicted at-sea mass-specific metabolic rate (DEE_PRED-S_ kJ d^−1^ kg^−1^).

### Approach 3

To investigate the accuracy of published activity-specific energy values in determining energy expenditure of free-ranging individuals, the time-activity budgets determined above were calculated using the following equation for each individual from published activity-specific energy values using the following equation:(9)
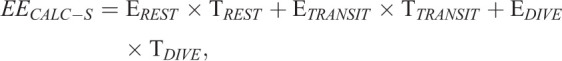
where EE_CALC-S_ is the total calculated at-sea energy expenditure (kJ), T*_i_* is the time spent (h) and E*_i_* is the activity-specific expenditure (kJ h^−1^) for each at-sea behaviour (sea-surface resting, transiting and diving). Published estimates of mass-specific energy expenditure for sea-surface resting and transit behaviour were derived from little penguins. As there was no such information for diving, a proxy was derived from a similar-sized (∼1 kg) seabird, the thick-billed murre (*Uria lomvia*), a species that also uses its wings for under-water propulsion (Table 3). The EE_CALC-S_ was converted to an estimate of calculated at-sea mass-specific metabolic rate (DEE_CALC-S_, kJ d^−1^ kg^−1^).

**Table 3.**
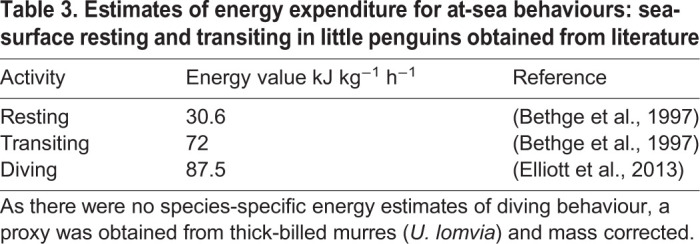
**Estimates of energy expenditure for at-sea behaviours: sea-surface resting and transiting in little penguins obtained from literature**

**Table 4.**
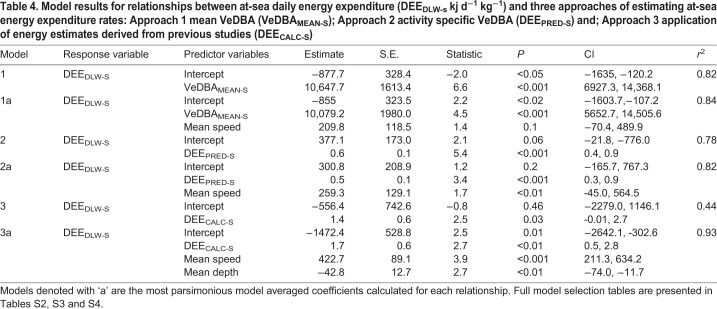
**Model results for relationships between at-sea daily energy expenditure (DEE_DLW-s_ kj d^−1^ kg^−1^) and three approaches of estimating at-sea energy expenditure rates: Approach 1 mean VeDBA (VeDBA_MEAN-S_); Approach 2 activity specific VeDBA (DEE_PRED-S_) and; Approach 3 application of energy estimates derived from previous studies (DEE_CALC-S_)**

The relationships between DEE_DLW-S_ and each metric of daily energy expenditure estimated from accelerometry (i.e. VeDBA_MEAN-S,_ DEE_CALC-S,_ DEE_PRED-S_) were determined using linear regression and the coefficient of determination (*r*^2^) was calculated. To establish whether these relationships could be improved, linear models were constructed to incorporate parameters that were likely to influence energy expenditure. Collinearity of predictor effects were assessed using Pearson's correlation test and parameters with a correlation >0.70 were removed from further analyses. The parameters modelled against DEE_DLW-S_ included: the foraging metrics mean horizontal travel speed (km h^−1^), total vertical distance travelled (km) and mean dive depth (m) and the metrics of daily energy expenditure estimated from accelerometry determined in Approaches 1–3 (i.e. VeDBA_MEAN-S_, DEE_CALC-S_, DEE_PRED-S_, respectively). Model selection was performed using the function *dredge* in the MuMIn package (Barton and Barton, 2015). The most parsimonious model was determined as having the lowest Akaike's Information Criterion corrected for small sample sizes (AICc) score and models with ΔAIC <4 are presented.

Normality was verified using Shapiro-Wilk tests and *t*-tests were performed to make group comparisons. Unless otherwise stated, results are presented as mean±s.e.

## Supplementary Material

Supplementary information
