# Supplementary material for: Validating accelerometry-derived proxies of energy expenditure using the doubly labelled water method in the smallest penguin species
Source: Biol Open. 2021 Apr 1;10(4):bio055475. doi: 10.1242/bio.055475 (PMC8034874; doi:10.1242/bio.055475)
Supplement: Supplementary information [file biolopen-10-055475-s1.pdf]

Table S1. Model results for relationship between at-sea daily energy expenditure (DEE_{DLW-s} $kJ·kg^{-1}·d^{-1}$) and mean speed ($km·h^{-1}$) and mean depth (m) per foraging trip.

Response variable	Predictor Variables	Estimate	S.E	Statistic	*P*	CI	r^2
DEE_{DLW-S}	Intercept	177.2	315.4	0.6	<0.5	-397, -737.4	
	Mean speed	543.3	131.3	3.5	<0.01	282.1, 887.8	0.64
	Mean depth	13.9	18.3	0.8	0.5	-27.3, 57.1	

Table S2. Full model selection table for model 1a. Models averaging was performed for candidate models with a delta < 4.

Model 1a									
Intercept	Horizontal distance	Mean Speed	VeDBA-mean-s	Vertical distance	K	logLik	AICc	delta	weight
-877.67	NA	NA	10647.7	NA	3	-59.66	129.32	0	0.6
-785.23	NA	209.77	8284.09	NA	4	-57.81	131.62	2.3	0.19
-740.84	NA	NA	8815.05	NA	4	-58.28	132.56	3.24	0.12
-856.12	NA	NA	10494.34	0	4	-59.64	135.28	5.96	0.03
-639.29	5.4	216.95	6290.8	NA	5	-55.42	135.84	6.52	0.02
63.07	10.18	409.94	NA	NA	4	-60.41	136.81	7.49	0.01
138.56	NA	542.31	NA	0.02	4	-60.94	137.88	8.56	0.01
263.7	NA	551.28	NA	NA	3	-64.15	138.3	8.98	0.01
-825.94	10.27	NA	8574.16	-0.01	5	-56.79	138.58	9.26	0.01
-628.27	NA	273.64	6647.6	0.01	5	-56.87	138.73	9.41	0.01
592.79	15.15	NA	NA	NA	3	-65.15	140.29	10.97	0
1278.77	NA	NA	NA	NA	2	-68.98	143.67	14.35	0
434.64	21.43	NA	NA	-0.02	4	-64.55	145.11	15.79	0
76.83	7.25	447.39	NA	0.01	5	-60.25	145.49	16.17	0
1129.38	NA	NA	NA	0.02	3	-67.85	145.71	16.39	0
-682.58	7.02	183.63	6600.11	0	6	-55.27	150.54	21.22	0

Table S3. Full model selection table for model 2a. Models averaging was performed for candidate models with a delta < 4.

Model 2a									
Intercept	Horizontal distance	Mean Speed	DEE$_{PRED-s}$	Vertical distance	K	logLik	AICc	delta	weight
195.07	8.32	NA	0.5	NA	4	-57.11	130.21	0	0.39
20.26	7.48	214.45	0.37	NA	5	-52.86	130.72	0.5	0.3
377.12	NA	NA	0.63	NA	3	-61.37	132.73	2.52	0.11
57.21	14.08	NA	0.49	-0.02	5	-54.09	133.18	2.96	0.09
143.4	NA	259.3	0.46	NA	4	-59.09	134.18	3.96	0.05
63.07	10.18	409.94	NA	NA	4	-60.41	136.81	6.6	0.01
382.32	NA	NA	0.59	0.01	4	-60.9	137.81	7.59	0.01
138.56	NA	542.31	NA	0.02	4	-60.94	137.88	7.66	0.01
263.7	NA	551.28	NA	NA	3	-64.15	138.3	8.09	0.01
96.04	NA	321.77	0.35	0.01	5	-56.71	138.43	8.21	0.01
592.79	15.15	NA	NA	NA	3	-65.15	140.29	10.08	0
1278.77	NA	NA	NA	NA	2	-68.98	143.67	13.45	0
1.4	10.47	156.29	0.4	-0.01	6	-52.07	144.13	13.92	0
434.64	21.43	NA	NA	-0.02	4	-64.55	145.11	14.89	0
76.83	7.25	447.39	NA	0.01	5	-60.25	145.49	15.28	0
1129.38	NA	NA	NA	0.02	3	-67.85	145.71	15.49	0

Table S4 Full model selection table for model 3a. Models averaging was performed for candidate models with a delta < 4.

Model 3a									
Intercept	DEE_{CALC-S}	Horizontal distance	Mean Speed	Vertical distance	K	logLik	AICc	delta	weight
-928.93	1.05	NA	460.86	NA	4	-59.83	135.65	0	0.31
-1863.8	1.87	24.38	NA	-0.06	5	-55.74	136.48	0.83	0.21
63.07	NA	10.18	409.94	NA	4	-60.41	136.81	1.16	0.18
138.56	NA	NA	542.31	0.02	4	-60.94	137.88	2.23	0.1
263.7	NA	NA	551.28	NA	3	-64.15	138.3	2.65	0.08
-709.71	0.75	6.61	395.52	NA	5	-57.18	139.36	3.71	0.05
592.79	NA	15.15	NA	NA	3	-65.15	140.29	4.64	0.03
-566.45	1.43	NA	NA	NA	3	-66.1	142.2	6.55	0.01
1278.77	NA	NA	NA	NA	2	-68.98	143.67	8.02	0.01
-287.56	0.83	10.99	NA	NA	4	-63.85	143.69	8.04	0.01
-660.93	0.77	NA	481.38	0.01	5	-59.43	143.86	8.21	0.01
434.64	NA	21.43	NA	-0.02	4	-64.55	145.11	9.46	0
76.83	NA	7.25	447.39	0.01	5	-60.25	145.49	9.84	0
1129.38	NA	NA	NA	0.02	3	-67.85	145.71	10.06	0
-1515.18	1.46	17.4	199.9	-0.04	6	-52.88	145.77	10.12	0
-691.41	1.55	NA	NA	0	4	-66.08	148.16	12.51	0
